# NMDA Receptor Signaling Mediates cFos Expression via Top2β-Induced DSBs in Glioblastoma Cells

**DOI:** 10.3390/cancers11030306

**Published:** 2019-03-05

**Authors:** Henrik Lutz, Thy Anh Nguyen, Juliane Joswig, Kerstin Rau, Bodo Laube

**Affiliations:** Neurophysiology and Neurosensory Systems, Technische Universität Darmstadt, Schnittspahnstrasse 3, 64287 Darmstadt, Germany; hennk@gmx.net (H.L.); thyanh@hotmail.de (T.A.N.); joswig@bio.tu-darmstadt.de (J.J.); kiki.rau@googlemail.com (K.R.)

**Keywords:** GBM, ionotropic glutamate receptor, topoisomerase IIβ, stimulus induced DSB, early response gene, NMDAR subunit GluN2B, radiotherapy, LN229, U-87MG, ifenprodil

## Abstract

The activation of Ca^2+^-permeable *N*-methyl-D-aspartic acid (NMDA) receptor channels (NMDARs) is crucial for the development and survival of neurons, but many cancers use NMDAR-mediated signaling as well, enhancing the growth and invasiveness of tumors. Thus, NMDAR-dependent pathways emerge as a promising target in cancer therapy. Here, we use the LN229 and U-87MG glioblastoma multiforme (GBM) cells and immunofluorescence staining of 53BP1 to analyze NMDAR-induced DNA double-strand breaks (DSBs), which represent an important step in the NMDAR signaling pathway in neurons by facilitating the expression of early response genes. Our results show that NMDAR activation leads to the induction of DSBs in a subpopulation of glioma cells. In a further analogy to neurons, our results demonstrate that the induction of DSBs in LN229 cells is dependent on the activity of topoisomerase IIβ (Top2β). Western blot analysis revealed that the inhibition of NMDARs, cAMP-responsive element binding transcription factor (CREB) and Top2β decreased the expression of the proto-oncogene cFos. Knockdown of Top2β with siRNAs resulted in a downregulation of cFos and increased the radiosensitivity of LN229 cells in clonogenic survival. We also observed impaired cFos expression upon NMDAR and Top2β inhibition in a primary GBM cell line, suggesting that NMDAR signaling may be widely used by GBMs, demonstrating the potential of targeting NMDAR signaling proteins for GBM therapy.

## 1. Introduction

Glutamate (Glu), the major excitatory neurotransmitter in the vertebrate central nervous system (CNS), operates via two types of receptors: Metabotropic glutamate receptors (mGluRs) and ionotropic glutamate receptors (iGluRs). Ca^2+^-permeable *N*-methyl-D-aspartic acid receptors (NMDARs) especially, as part of the iGluR family, have gained particular attention because of their crucial roles in brain development, synaptic plasticity and memory formation, but also in neurotoxicity [1,2]. NMDARs are composed as tetrameric assemblies with two essential GluN1 subunits and varying contributions of two GluN2A-D subunits or possibly also GluN3 subunits [3,4]. The specific expression of the subunits differs between brain regions as well as during development and determines the functional specificity of the NMDAR subtypes [5]. During brain development, NMDAR-mediated Ca^2+^-influx regulates fundamental cellular processes, including the proliferation, migration and survival of neurons [6], playing a lifelong essential role in the formation of synaptic plasticity [2]. NMDAR-signaling acts by activating two major downstream signaling pathways: The Ca^2+^/calmodulin kinase (CaMK-II/IV) pathway and the mitogen-activated protein kinase (MAPK) pathway [7]. In response to Glu upon neuronal activity, both NMDAR-dependent pathways activate the cAMP-responsive element binding transcription factor (CREB) and induce the expression of several transcription factors, so-called early response genes (ERGs). These ERGs encode transcription factors (for example, c-Fos and EGR1), which promote the induction of further neuronal activity-regulated genes (for example, the brain derived neurotrophic factor, BDNF), which are crucial for neuronal growth, survival and synaptic plasticity [5,8]. To facilitate transcription, it has been shown that CREB and RNA polymerase II rest at the gene site of ERGs. In 2015, Madabhushi et al. [9] demonstrated that upon NMDAR activation in neurons, the topoisomerase IIβ (Top2β) gets activated and induces DNA double strand breaks (DSBs) in the promotor region of the ERGs. These DSBs dissolve the topological constraints to enhancer-promoter interactions and therefore stimulate the rapid gene transcription of ERGs via the resting RNA polymerase II. Although Top2β-mediated DSBs are needed for functional NMDAR signaling, the misrepair of such DSBs is considered to promote neurodegenerative diseases and also carcinogenesis [9,10,11].

In addition to neurons, functional NMDARs have been found in a wide variety of tumor types and cancer cell lines, indicating NMDAR signaling in cancerous cells and highlighting NMDARs as a promising therapeutic target in cancer therapy [12,13]. In human tumors, these Ca^2+^-permeable receptors may play a regulative role beyond the traditional role of NMDARs in excitatory neurotransmission and synaptic plasticity. It has already been shown that NMDAR antagonists reduce both tumor growth and invasiveness across many different cancers and have anti-tumoral effects when used in various xenograft tumors [14,15,16,17,18,19,20]. In addition, higher expression levels of NMDARs in tumors are associated with a worse prognosis for cancer patients [15,16,21,22]. The NMDAR signaling pathways used by cancer cells are thought to be similar to neurons and have been implicated in tumor progression [14,22]. For example, in lung adeno-carcinoma and glioblastoma multiforme (GBM) cells, it has been revealed that NMDAR antagonists inhibit the ERK1/2 and CREB pathways, respectively [19,23]. The role of NMDAR signaling in cancer is strengthened in respect to the findings that both GBM cells and non-CNS cancers secrete high levels of glutamate [22,24,25,26], which promotes tumor growth, as well as the survival and migration of the tumor cells [27]. The main release of Glu by cancer cells is mediated by the highly expressed system X_c_^−^ cystine/glutamate (Cyss/Glu) antiporter [28,29]. Inhibition of the system X_c_^−^ with the drug sulfasalazine (SAS) leads to a decreased tumor growth and reduced tumor formation in xenografted mice [30,31], further demonstrating the importance of Glu-activated NMDARs as a regulator of cancer cell growth and tumor progression. 

The precise mechanisms underpinning the NMDAR-mediated cellular effects in cancers are, compared to neuronal NMDARs, poorly understood, but there is a developing consensus that they may play a therapeutic role in cancer treatment [12]. Thus, we investigated if a particular step of neuronal NMDAR signaling, which might be interesting for cancer therapy, was analogous with glioblastoma cells: The Top2β mediated gene activation via induction of DSBs. Therefore, we analyzed the contribution of iGluRs and Top2β in the induction of DSBs and the expression of the ERG cFos in the LN229 glioblastoma cell line. We found that Glu induces transient DSBs in a subpopulation of cells, which are mainly mediated through NMDAR activation and that the induction of Glu-depended DSBs requires Top2β activity. Thirdly, we found that NMDAR signaling regulates cFos expression with a strong effect on the radioresistance of the glioblastoma cells. Concluding, our study contributes to a novel conceptual insight in NMDAR-mediated signaling as a new therapeutic area for treating cancer cells, which may help to develop adjusted treatments for cancers by understanding the mechanistic contributions and pathologic significance of NMDAR activation in tumors.

## 2. Results

### 2.1. Glutamate Induces DSBs in LN229 and U-87MG GBM Cells

**Figure 1:**
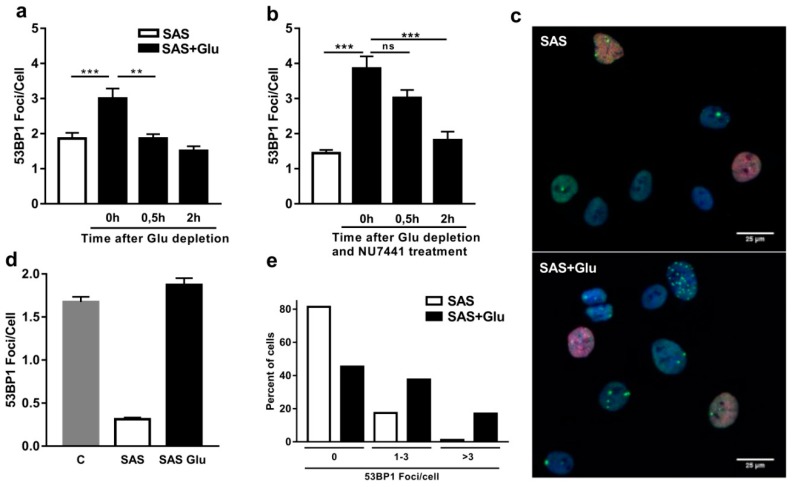
Glutamate (Glu) induces transient double-strand breaks (DSBs) in LN229 cells. (**a**) Overnight treatment with 1 mM Glu increased the mean number of 53BP1 foci/cell in non-S-phase LN229 cells cultivated with 250 µM sulfasalazine (SAS). Depletion of Glu lead to a reduction of foci to a basal level after 0.5 h (*n* = 3; 40 cells/n, bar graphs show the mean of all single values). (**b**) The repair of 53BP1 foci was delayed for 2 h when 1 µM NU7441 was given at the time point of Glu depletion, indicating a repair by non-homologous end joining (NHEJ) (LN229 cells treated with 250 µM SAS and 1 mM of Glu overnight. *n* = 3; 40 cells/n; bar graphs show the mean of all single values). (**c**) Representative immunofluorescence staining of LN229 cells treated with 250 µM SAS or 250 µM SAS and 1 mM of Glu. Green = 53BP1, red = EdU, blue = Hoechst33342. Note that the LN229 cells show a heterogeneous distribution of 53BP1 foci after Glu treatment (Scale bar: 25 µm). (**d**,**e**) High content counting of 53BP1 foci in LN229 cells treated with 250 µM SAS or 250 µM SAS/1 mM of Glu or untreated (*n* = 1; >1500 cells/n). (**d**) Cells treated with Glu and untreated cells show a higher number of 53BP1 foci/cell (>1500 cells). (**e**) Distribution of 53BP1 foci within the cell population. About 80% of the cells have no foci when treated with SAS but the number of cells without foci decreased in the presence of Glu. Glu treatment increased the low (1–3) and high (>3) numbers of foci in LN229 cells, indicating differential responses of subpopulations (>1500 cells/n). (All error bars show SEM. Mann–Whitney Test for statistics; *p* > 0.05 (ns), *p* ≤ 0.05 (*), *p* ≤ 0.01 (**), *p* ≤ 0.001 (***)).

**Figure 2:**
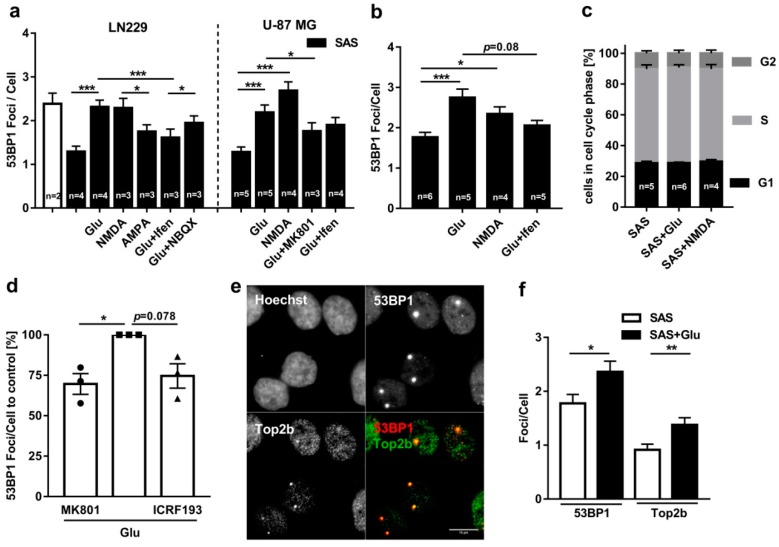
Role of *N*-methyl-D-aspartic acid receptors (NMDARs) and Top2β on Glu-induced double-strand breaks (DSBs). (**a**) LN229 (left) and U-87MG (right) cells were treated with 250 µM SAS and 1 mM of Glu, 100 µM NMDA, 100 µM AMPA, 1 mM of Glu/20 µM ifenprodil, 1 mM of Glu/100 µM NBQX, 1 mM Glu/20 µM MK801 or kept untreated overnight. The 53BP1 foci per cell of the G1/G2 cells was counted. The number of 53BP1 foci/cell increased with Glu, as well as with NMDA, and reached the same level as the untreated cells. AMPA induced a significantly lower amount of foci than NMDA in the LN229 cells. Ifenprodil and MK801 led to a strong decrease of foci (n indicated in bar diagrams; 50 cell/n; bar graphs show the mean of all single values; error bars show SEM; Mann–Whitney test). (**b**) Analysis of 53BP1 foci/cell in G1 phase LN229 cells treated with 250 µM SAS and 100 µM NMDA, 1 mM Glu, 1 mM Glu/20 µM ifenprodil or untreated overnight. There was a significant increase of 53BP1 foci/cell with NMDA or Glu but no significant reduction with 20 µM ifenprodil, as compared to the Glu treatment. (n indicated in bar diagrams, 50 cell/n, bar graphs show the mean of all single values; error bars show SEM; Mann–Whitney test). (**c**) Cell cycle analysis of LN229 cells treated with 250 µM SAS and 1 mM Glu or 100 µM NMDA overnight. Treatment with Glu or NMDA did not change the cell cycle distribution. Phases were gated by EdU and the Hoechst 33342 signal. (n indicated in bar diagrams; at least 1000 cells/n; error bars show SEM; students *t*-test). (**d**) Analysis of 53BP1 foci/cell in G1 phase LN229 cells treated with 1 mM Glu and 20 µM MK801 or 100 nM of the Top2β inhibitor ICRF193. MK801 led to a significant decrease in the relative amount of 53BP1 foci/cell. ICRF193 showed a similar, but not significant effect. (*n* = 3; 50cells/n; error bars show SEM; one sample *t*-test). (**e**) Immunofluorescence staining of Hoechst 33342, 53BP1 (red) and Top2β (green) in LN229 cells. Top2β formed foci which co-localized with 53BP1 foci (Scalebar: 25 µm). (**f**) LN229 cells were treated with 250 µM SAS with/without 1 mM Glu and 53BP1 and Top2β foci/cell were counted in G1/G2 cells. Glu increased the number of 53BP1 and Top2β foci/cell (*n* = 2; 40 cells/n; bar graphs show the mean of all single values; error bars show SEM; Mann–Whitney test). (*p* > 0.05 (ns), *p* ≤ 0.05 (*), *p* ≤ 0.01 (**), *p* ≤ 0.001 (***)).

It has been shown in neurons that Glu can induce double-strand breaks (DSBs) upon the specific activation of *N*-methyl-D-aspartic acid receptors (NMDARs) [9]. To consider whether Glu is also able to induce DSBs in NMDAR expressing LN229 and U-87MG cells [19], we quantified the number of DSBs through the immunostaining of 53BP1 foci after Glu treatment. In the first experiment, the LN229 and U-87MG cells were treated with 250 µM sulfasalazine (SAS), which inhibits the endogenous release of Glu by the system X_c_^−^ antiporter [31]. The number of 53BP1 foci in non-S-phase cells was counted (EdU negative). The SAS treated LN229 and U-87MG cells showed a mean number of 1.9 ± 0.2 and 1.3 ± 0.1 53BP1 foci/cell, respectively (Figure 1a). In contrast, cells treated with 1 mM Glu showed a significantly increased number of 53BP1 foci (LN229: 3.0 ± 0.3; *p* < 0.0001; U-87MG: 2.2 ± 0.2; *p* < 0.001; Figure 1a and Figure 2a), indicating that Glu induces DSBs in both LN229 and U-87MG cells, similar to what is described in neurons. Remarkably, after 0.5 h depletion of Glu decreased the 53BP1 foci to 1.9 ± 0.1 (*p* = 0.0019), a number of foci similar to the SAS treated LN229 cells without Glu treatment (Figure 1a). Thus, our results indicate that the Glu-induced DSBs are rapidly repaired after Glu depletion. To see whether the decrease of 53BP1 foci in our non-S-phase cells indeed reflects a repair of DSBs, we performed the same experiment in the presence of the DNA-PK_cs_ inhibitor NU7441 (1 µM) (Figure 1b). Half an hour after Glu depletion, the number of 53BP1 foci was no longer decreased upon NU7441 treatment (3.0 ± 0.2). Only after 2 h was a decrease to 1.8 ± 0.2 foci/cell found, indicating a delayed repair of Glu-induced DSBs upon DNA-PK_cs_ inhibition (Figure 1b). These results demonstrate that transiently induced 53BP1 foci in LN229 cells represent DSBs, likely repaired by non-homologous end joining (NHEJ). Interestingly, we realized differences in the number of DSBs within individual LN229 cells (Figure 1c) and hypothesized that only a fraction of LN229 cells respond to Glu treatment. Therefore, we chose to analyze 53BP1 foci in a higher number of cells using automated, high-content microscopy. Again, the cells were treated with 250 µM SAS, with or without Glu, or left untreated. At least 1500 non-S-phase cells were imaged and the 53BP1 foci were automatically counted. Similar to our first results, the number of foci per cell in the SAS treated cells increased after Glu treatment (1.9 ± 0.1 vs. 0.3 ± 0.02) (Figure 1d). Next, we analyzed the distribution of the number of foci per cell within the LN229 cell population. Eighty-one percent of all cells treated with SAS had no foci, and 17.4% showed between 1 and 3 foci (Figure 1e). After Glu treatment, 45.4% of all cells showed no foci, indicating that only 36% of the cells specifically reacted to Glu by DSB induction. Furthermore, our result also indicates that almost half of the cells did not respond to Glu treatment at all. The proportion of cells with 1–3 foci per cell increased to 37.6% for Glu treated cells, and the number of cells with higher amounts (>3 foci/cell) of DSBs increased as well (17.0%). Thus, our results revealed the induction of higher amounts of transient DSBs by glutamate only in a subpopulation of LN229 cells.

### 2.2. DSB Induction is Dependent on NMDARs and Top2β

To confirm whether the Glu-induced DSBs in the LN229 and U-87MG cells are indeed mediated by calcium permeable NMDARs and not by other subtypes of iGluRs, we analyzed the number of 53BP1 foci after the application of specific agonists and antagonists of AMPARs and NMDARs. Therefore, we inhibited the endogenous release of glutamate with 250 µM SAS, treated LN229 cells with 1 mM of Glu, 100 µM NMDA or 100 µM AMPA overnight and quantified the 53BP1 foci in the non-S-phase cells (Figure 2a). NMDA treatment led to a number of 53BP1 foci (2.3 ± 0.2), comparable to the Glu treated cells, whereas the addition of AMPA showed a significantly lower number of foci when compared to the NMDA treatment (1.8 ± 0.2, *p* = 0.016, Mann–Whitney Test (MWT)). To further verify the specificity of the iGluR mediated induction of DSBs, we treated LN229 cells with Glu in the presence of the specific AMPAR antagonist NBQX (100 µM) or the NMDAR antagonist ifenprodil (20 µM). As shown in Figure 2a, adding ifenprodil resulted in a highly significant decrease in 53BP1 foci (1.6 ± 0.2, *p* < 0.001) when compared to the Glu treatment alone, whereas adding NBQX led to a significant lower decrease of the number of foci compared to ifenprodil (1.95 ± 0.16, *p* = 0.026). In the U-87MG cells, the number of 53BP1 foci/cell significantly increased to 2.2 ± 0.2 (*p* < 0.001) when treated with Glu, compared to the cells only treated with SAS (1.3 ± 0.1) (Figure 2a). To check whether the foci induction in U-87MG cells is also dependent on NMDAR activation, we treated the cells with NMDA (100 µM), which significantly increased the number of foci/cell to 2.7 ± 0.2 (*p* < 0.001). For validation of the NMDAR dependent effect, the cells were treated with the specific NMDAR inhibitors MK801 (20 µM) and ifenprodil (20 µM). MK801 significantly decreased the number of foci/cell to 1.8 ± 0.2 (*p* = 0.014) (Figure 2a). The significant decrease in Glu-induced 53BP1 foci in the presence of specific NMDAR antagonists and the higher number of 53BP1 foci after NMDA treatment led us to the assumption that Glu-induced DSBs are mainly mediated via NMDARs in both LN229 and U-87MG cells. Madabhushi et al. [9] showed in 2015 that these NMDAR-induced DSBs are mediated by Top2β and have a regulative function on the transcription of several genes in neurons. Based on this comparability, we questioned whether NMDAR-induced DSBs might have a similar regulatory role in tumor cells. Since most transcriptional activity happens in the G1 phase of the cell cycle [32,33], we expected that our Glu-induced DSBs should be mainly observed in G1 phase cells. Therefore, we treated the LN229 cell with 250 µM SAS and 100 µM NMDA, 1 mM of Glu or 1 mM of Glu and 20 µM ifenprodil and counted the number of 53BP1 foci in G1 phase cells only (Figure 2b). NMDA treatment, as well as the addition of Glu, significantly increased the number of 53BP1 foci from 1.8 ± 0.7 to 2.3 ± 0.2 (*p* = 0.013) and 2.8 ± 0.2, respectively (*p* < 0.001), indicating that Glu-mediated DSBs occur predominantly in the G1 phase. However, to rule out whether the differences in foci numbers were caused by changes in the cell cycle distribution within the LN229 cell population, we analyzed the cell cycle distribution after treatment with Glu and NMDA in the presence of SAS. Neither Glu nor NMDA treatment revealed differences in the cell cycle distribution within the LN229 cell population (Figure 2c). So far, our results indicate that Glu-induces NMDAR-mediated DSBs in LN229 cells, comparable to the mechanisms previously described in neurons. To test whether inhibiting the endonuclease activity of Top2β and/or NMDAR activity affects Glu-induced DSBs to a similar extent, we counted the number of 53BP1 foci in G1 cells in the presence of the specific Top2β inhibitor ICRF193 (100 nM) or the specific NMDAR blocker MK801 (20 µM). Strikingly, compared to cells treated with Glu, inhibition of both NMDAR and Top2β revealed a similar decrease of 53BP1 foci to 69.6 ± 6.4% and 74.6 ± 7.6%, respectively (Figure 2d). However, statistical analyses revealed only for MK801 treated cells a significant decrease (MK801: *p* = 0.042; ICRF193: *p* = 0.078), which could be attributed to the intrinsic DSB induction by ICRF193 itself [34]. To further analyze the role of Top2β in the induction of DSBs in LN229 cells, we performed the double immunostaining of Top2β and 53BP1 after Glu treatment. Remarkably, Top2β formed foci-like structures which co-localized with the 53BP1 foci (Figure 2e), indicating that Top2β is accumulated in the vicinity of, or even associated with, Glu-induced DSBs in LN229 cells. To analyze whether Top2β foci in LN229 cells can be similar induced as 53BP1 foci upon NMDAR activation, we analyzed the number of Top2β and 53BP1 foci in the presence of SAS (250 µM), with and without Glu (Figure 2f). Strikingly, treatment with Glu (1 mM) revealed a significant increase in the number of Top2β foci from 0.9 ± 0.1 to 1.4 ± 0.1 (*p* = 0.009), which is more significant than the increase of 53BP1 foci (1.8 ± 0.2 to 2.4 ± 0.2, *p* = 0.034). Collectively, our results show that the activation of NMDARs by Glu induces Top2β-mediated DSBs that are independent of cell cycle progression in the glioblastoma cell line LN229. Furthermore, our data indicate a comparable role of NMDAR-mediated signaling for the induction of DSBs in both neurons and glioblastoma cells.

### 2.3. NMDAR Signaling Regulates cFos Expression and Promotes Radioresistance in LN229 Cells

**Figure 3:**
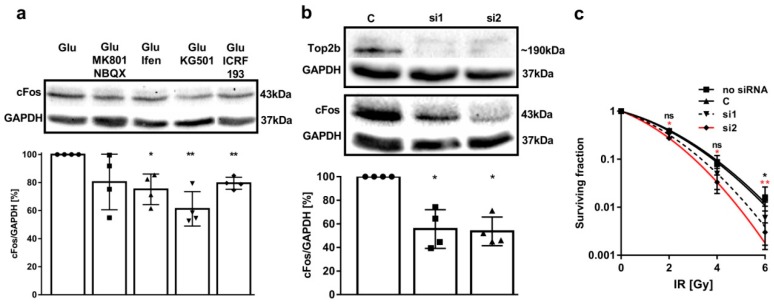
NMDAR signaling regulates cFos expression and promotes radioresistance in LN229 cells. (**a**) Relative cFos/GAPDH expression was analyzed through western blotting. Overnight inhibition of NMDARs with 20 µM ifenprodil, as well as inhibition of CREB with 25 µM KG501 and Top2β with 1 µM ICRF193, led to a significant decrease in cFos expression. Relative cFos expression is highly variant upon the inhibition of NMDARs and AMPARs with 20 µM MK801 and 100 µM NBQX. (*n* = 4; error bars show SD; one sample *t*-test). (**b**) Successful knockdown of Top2β with two different siRNAs led to a significant downregulation of cFos. Relative cFos/GAPDH expression was analyzed through western blotting. (*n* = 4; error bars show SD; one sample *t*-test). (**c**) Clonogenic survival of LN229 cells treated with 1 mM Glu and transfected with two different siRNAs against Top2β or transfected without RNA and irradiated with 0, 2, 4 and 6 Gy X-ray. Diagram shows fitted data. siRNA2 significantly reduces the survival starting at 2 Gy. siRNA1 shows an intermediate effect. (*n* = 3, each experiment was performed as triplet; error bars show SD; student’s *t*-test). (*p* > 0.05 (ns), *p* ≤ 0.05 (*), *p* ≤ 0.01 (**), *p* ≤ 0.001 (***)).

Our results demonstrate that the constitutive release of Glu induces NMDAR-dependent DSBs in LN229 and U-87MG cells, indicating similar NMDAR signaling pathways in glioblastoma multiforme (GBM) cells and neurons. To investigate if NMDAR-induced DSBs are also capable of regulating the transcription of genes in GBM cells, we chose to analyze the impact of NMDAR signaling and Top2β activity on the expression of the early response gene (ERG) cFos, a protein which is expressed upon the Top2β-mediated DSB-induction in neurons [9] and has been implicated in GBM malignancy [35]. Thus, we inhibited iGluR signaling with 20 µM MK801/100 µM NBQX and quantified cFos expression in LN229 cells by western blotting (Figure 3a). However, the variance of the results was quite high, reaching from no effect to almost a 50% decrease, with a mean reduction of cFos expression of 80 ± 20% (*p* = 0.142). Interestingly, the GluN2B specific antagonist ifenprodil (20 µM) significantly reduced the expression of cFos to 75 ± 11% (*p* = 0.02), which confirms the prominent role of the GluN2B subunit in LN229 cells [19]. Accordingly, we inhibited two key proteins downstream in the NMDAR signaling cascade: CREB and Top2β, with 25 µM KG501 and 1 µM ICRF193, respectively (Figure 3a). CREB inhibition significantly decreased the relative expression of cFos to 61 ± 12% (*p* = 0.008) and Top2β inhibition led to a downregulation of cFos to 80 ± 4% (*p* = 0.002). These results show that NMDAR signaling regulates cFos expression in LN229 cells and support our idea of Top2β-mediated DSBs regulating gene expression in GBM. 

Since cFos expression is correlated with radioresistance in GBM cells [35], we intended to verify whether silencing Top2β would also increase the sensitivity of LN229 cells to radiation. Thus, we performed a siRNA knockdown of Top2β, which resulted in a pronounced decrease of Top2β protein expression (Figuire 3b). Both siRNA treatments also decreased cFos expression by about 50% (si1 56 ± 16%, *p* = 0.012; si2 54 ± 6%, *p* = 0.005, Figure 3b), confirming the importance of Top2β for cFos expression in LN229 cells. Next, we determined the effect of the two siRNA-mediated Top2β knockdown on radio sensitivity by clonogenic survival upon irradiation with 0 Gy, 2 Gy, 4 Gy and 6 Gy X-rays (Figure 3c). The survival curves show that the Top2β knockdown with siRNA2 significantly reduced the survival of the cells already at 2 Gy (*p* = 0.014), while the siRNA1 showed an intermediate effect. These results highlight the specific role of NMDAR signaling on Top2β-mediated transcriptional activity for radioresistance in the LN229 GBM cell line, indicating Top2β as a potentially therapeutic target for GBMs. 

### 2.4. NMDAR Dependent cFos Regulation in a Primary GBM Cell Line

**Figure 4:**
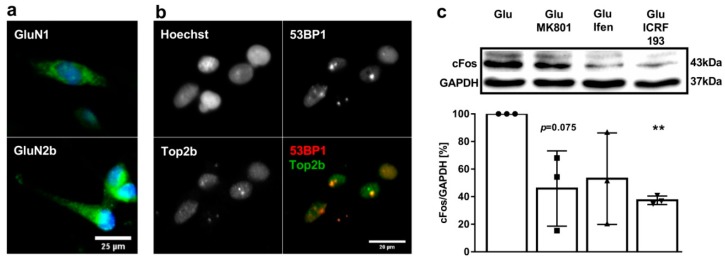
NMDAR signaling impacts cFos expression in primary glioblastoma multiforme (GBM) cells. (**a**) Immunofluorescence staining of the GluN1 and GluN2B subunits of the NMDAR in the G1702 cells. Notably, GluN2B subunits are localized at the end of cellular protrusions (GluN1/GluN2B = green, Hoechst 33342 = blue; scale bar: 25 µm). (**b**) Immunofluorescence staining of 53BP1 (red) and Top2β (green) in G1702 cells. Top2β and 53BP1 form foci which partly co-localize (scale bar: 20 µm). (**c**) Relative cFos/GAPDH expression in G1702 cells treated with 1 mM Glu and 20 µM MK801, 20 µM ifenprodil or 1 µM ICRF193 overnight, analyzed through western blotting (error bars show SD, *n* = 3, one sample *t*-test, *p* > 0.05 (ns), *p* ≤ 0.05 (*), *p* ≤ 0.01 (**), *p* ≤ 0.001 (***)).

To verify whether the NMDAR-mediated transcriptional increase of ERGs also occurs in primary GBM cells isolated from human tumor samples, we first analyzed the functional expression of iGluRs in the primary GBM cell line G1702 (kindly provided by Prof. Donat Kögel, Frankfurt University Hospital, Frankfurt am Main, Germany) through immunohistochemistry and patch-clamp recording. The immunostaining and electrophysiological analyses of the G1702 cells showed a robust expression of GluN1 and GluN2B NMDAR subunits, with Glu-mediated mean currents of 42 ± 17 pA upon application of 1 mM Glu/100 µM glycine in 46% of the G1702 cells tested (*n* = 15; Figure 4a). Next, we checked the co-localization of Top2β at the site of DSBs indicated by 53BP1 foci upon Glu treatment (Figure 4b). As shown by immunostaining, Top2β forms foci which co-localize with 53BP1 foci, indicating that Top2β, similar to our finding in the LN229 cells, is also mediating the induction of DSBs in the G1702 cells. Finally, to analyze the effect of NMDAR and the Top2β antagonists on cFos expression, we treated the G1702 cells with 1 mM Glu and 20 µM MK801, 20 µM ifenprodil or 1 µM ICRF193 and quantified cFos expression through western blotting (Figure 4c). For the MK801 treatment, we observed a mean cFos expression of 46 ± 27% when compared to the Glu treated cells (*p* = 0.075). The mean expression of cFos after ifenprodil treatment was 53 ± 33% that of the control (*p* = 0.134). Although the mean expression of cFos indicates an even stronger downregulation than in the LN229 cells, high variances do not allow us to determine significant changes in cFos. In contrast, the treatment with the Top2β antagonist ICRF193 decreased the mean expression of cFos significantly to 37 ± 3% in the G1702 cells (*p* < 0.001), underlining the striking importance of the Top2β on cFos expression in GBM cells. Thus, by using an established GBM cell line and primary cells isolated from a human tumor sample, we can reproduce a NMDAR-dependent induction of cFos expression by using Top2β as a common phenomenon in GBM cells, which might constitute a key aspect of glioblastoma biology and therapy, including tumorigenicity and therapeutic resistance.

## 3. Discussion

In this study, we demonstrate the prominent role of NMDARs in the induction of DSBs in LN229 cells and the impact of the Top2β on this process. Inhibiting CREB- and Top2β-activity shows that NMDAR signaling regulates cFos transcription in GBM cells, with a high impact on radioresistance. The involvement of NMDARs, CREB and Top2β in the transcriptional regulation of cFos indicates a similar regulatory mechanism in GBM cells and neurons. Thus, to our knowledge, this study is the first which demonstrates that NMDAR-mediated and Top2β-dependent DSBs promote radioresistance in a GBM cell line. Top2β-dependent transcriptional regulation of the early response gene cFos in the LN229 and primary GBM cells suggests that the neuronal Top2β-dependent signaling pathway maybe a common phenomenon responsible for promoting malignant growth in glioma cancers. However, the downstream effectors of NMDARs and how their activity modulates GBM physiology by the transcriptional regulation of ERGs will be a promising subject for further studies exploring the clinical benefits of NMDAR and Top2β antagonists.

**Figure 5:**
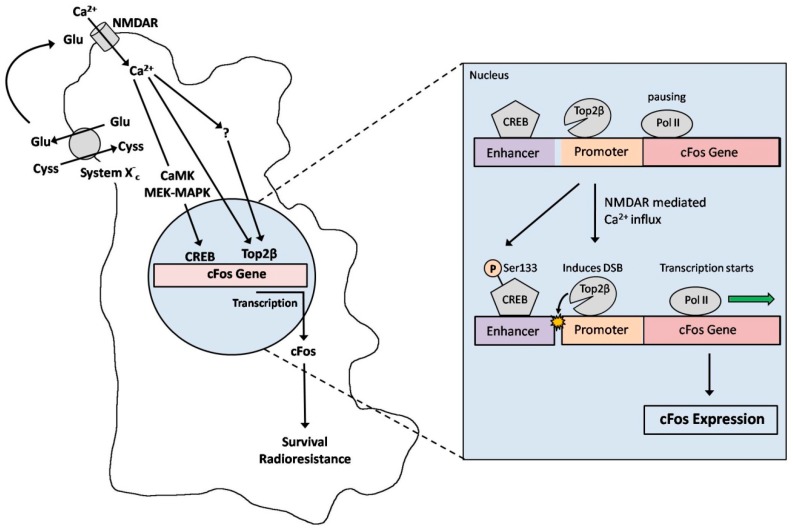
Schematic model of NMDAR-mediated signaling in GBM cells. GBM cells secrete Glu via the Glu/Cyss co-transporter system X_c_^−^. Glu activates NMDARs, which mediate Ca^2+^ influx into the cell. The increased Ca^2+^ concentration leads to the activation of the cAMP-responsive element binding transcription factor (CREB) at the enhancer/promoter region of the early response genes (cFos), probably via the phosphorylation of Ser133 by Ca^2+^/calmodulin-dependent protein kinases (CaMK) or the MEK-MAPK pathway. Top2β is enriched at CREB binding sites [9] and activated by Ca^2+^ (or via an unknown signaling pathway, or both) indicating an overlap of Top2β activity with CREB-mediated gene regulation. Upon activation, Top2β induces a DSB in the promoter region of the cFos gene, reducing the topological constraints of the DNA, which allows the pausing of Pol II to start transcription. Finally, the expression of cFos improves the survival and radio resistance of GBM cells.

The relevance of NMDAR-mediated signaling for GBM biology, including tumorigenicity, invasion and therapeutic resistance has been already demonstrated recently [19,22,36]. By now, it has been shown that both GBM cells and non-CNS cancers secrete high levels of glutamate [22,24,25,26] and that NMDARs in tumor cells mediate glutamatergic signaling via the ERK1/2 and CREB pathways (see Figure 5) [19,23]. In the neuronal pathway, activation of NMDARs leads to the phosphorylation of CREB, which mediates the transcription of ERGs, such as EGR1 and cFos. Remarkably, some ERGs are proto-oncogenes and their sustained expression can have profound effects on cellular growth. It has been shown that there is enrichment for specific transcription factor binding sites within the regulatory regions of ERGs, including CREB binding sites, indicating conserved mechanisms of transcriptional regulation within neurons and tumor cells. However, it should be mentioned that the NMDAR signaling pathways in the CNS are highly complex, regulated at multiple levels to allow fine-tuning of neuronal activities which might be differentially used in cancer cells [7,12]. Nevertheless, we have now found in several experiments that an effective part of NMDAR signaling in neurons is also present in LN229 cells: The induction of DSBs by Top2β upon Glu treatment by specific activation of NMDARs (see Figure 5). This is supported by our finding that NMDAR activation increased the number in Top2β foci co-localizing with 53BP1 foci, which indicates specific Top2β activity at DSB sites upon NMDAR stimulation. Thus, our results conceptually extend previous studies, describing the activity of NMDAR signaling in several cancer types, as well as the putative benefits of its pharmacological inhibition. Interestingly, Top2β induces DSBs in neurons in the promoter region of cFos and other ERGs upon NMDAR activation [9]. These DSBs are needed to start gene transcription. Since ERG expression like cFos and EGR1 has been reported to correlate with radioresistance and poor prognosis in malignant glioma [35,37], we wondered if NMDAR signaling was also capable of regulating Top2β-dependent cFos expression in GBM cells. Our work clearly confirms NMDAR-dependent cFos expression in LN229 cells upon the inhibition of NMDARs, CREB and Top2β (summarized in Figure 5). Interestingly, Top2β knockdown led to a stronger downregulation of cFos than inhibiting NMDARs. Since transcription-induced DSBs mediated by Top2β are not restricted to NMDAR signaling [11,38], the Top2β knockdown possibly deregulates additional gene transcription, resulting in a more pronounced cFos downregulation. This is in line with our finding that the inhibition of Top2β did not reflect the high variance of NMDAR inhibition, which indicates that Top2β activation might not be exclusively restricted to NMDAR signaling. However, we found that the GluN2B specific antagonist ifenprodil robustly decreased cFos expression, underlining a specific role of the GluN2B subunit in NMDAR-mediated signaling in GBM. The impact of GluN2B signaling has already been reported in GBM cells and other cancers [15,19,21,22]. In this context, what was unexpected was that the decrease in cFos expression by ifenprodil was more pronounced than in the MK801 treatment. Since MK801 blocks all NMDARs, it should at least be as effective as ifenprodil. The reason why this is not the case could be because of the differential activation of mitogen activated protein (MAP)-kinase signaling by the GluN2A or GluN2B bearing receptors, leading to a diverse cellular outcome depending on the NMDAR subunit expression [39]. Another possibility is the relatively low stability of MK801 under cell culture conditions, with a half-life of about an hour [40]. Our concentration of 20 µM MK801 should be sufficient to block NMDARs, but overnight incubations at 37 °C could possibly decrease the effective amount of inhibitor due to degradation. Other publications showed MK801 to be effective in ranges of up to 50 µM in cellular assays of different cancer cell lines [14], or even used in concentration up to 300 µM [15], indicating the need for higher concentrations under cell culture conditions. Even so, ifenprodil (and therefore the GluN2B subunit), seems to play an important role in NMDAR signaling in LN229 GBM cells. In summary, although the specific impact of distinct NMDAR subunits on Top2β activity is still enigmatic, the importance of Top2β for NMDAR-expressing GBM cells has been proven by us through the radio sensitizing effect of Top2β knockdown in the clonogenic survival assay, which identifies Top2β as a feasible therapeutic target in tumor therapy.

In cultured primary neurons stimulated with NMDA, Chromatin ImmunoPrecipitation DNA-Sequencing ChIP-seq with antibodies against H2AX and PCR-based assays suggest that stimulus induced DSBs form within 21 genomic loci [9]. Remarkably, we found that the mean increase of DSBs in LN229 upon NMDAR activation seems to be relatively small (2–4 foci). We attribute the lower mean number of Glu-induced DSBs to our finding that less than half of the LN229 and primary GBM cells express functional iGluRs ([19] and this study) and thus a majority of the cells should not be able to respond to Glu with the induction of DSBs. Consistent with this idea, the fraction of LN229 cells that specifically respond to Glu treatment with the induction of DSBs (36%) closely matches the fraction of cells with functional iGluRs (40%), as determined by electrophysiological measurements [19]. Our results from the primary G1702 GBM cells indicate that NMDAR signaling is not restricted to an immortalized cell line. Similar to the situation in LN229 cells, MK801 and ifenprodil both affected cFos expression in the G1702 cells, but with a higher variance. When grown in monolayers, only 46% of all G1702 cells showed a functional expression of iGluRs, which, however, might differ when the cells are grown as spheres. Since NMDARs are thought to be highly expressed in the marginal zone of the tumor [22], the expression of NMDARs may depend on the size of the spheres and vary under different culture conditions. Nevertheless, although cultured tumor cell lines from glioblastomas may not fully reflect the genotypes and phenotypes of the respective primary tumors, inhibition of Top2β in G1702 cells led to a reliable downregulation of cFos, which was remarkably more pronounced than in the LN229 cells. This result indicates that at least a subset of our primary cells use the signaling pathway found in LN229 cells. Furthermore, these results may also indicate the existence of a subpopulation within GBM cells with distinct properties, which could be characterized by the expression of functional iGluRs. Interestingly, GBM subpopulations expressing iGluRs have been correlated with brain tumor initiating cells [41], and especially the expression of the GluN2B subunit shows prognostic relevance in GBM tumors [22]. Furthermore, Top2β has also been reported to be overexpressed in GBM tumor initiating cells [42]. These findings suggest that iGluR expressing GBM cells, which are able to induce DSBs via Top2β, might represent a subpopulation of cells with high therapeutic relevance.

Exploiting transcriptional DSBs for cancer therapy has already been suggested [38], for example by inducing DSBs through the corresponding receptors and simultaneous administration of DNA repair inhibitors or topoisomerase poisons, such as etoposide. Our results show that NMDAR and Top2β activation may be used in this way to induce DNA damage in distinct GBM cells, possibly in adjuvant radiotherapy. In contrast, another publication ascribes Top2β a role in DSB repair [42], which would explain the radiosensitizing effect of Top2β inhibition, but our finding that inhibition of the Top2β endonuclease activity with ICRF193 led to a decreased number of foci in LN229 cells indicates a role of Top2β in the induction of DSBs rather than the repair. However, it is assumed that Top2β can both ensure and endanger genome integrity. Inhibition of DNAPK_cs_ demonstrates that the Top2β-induced DSBs are likely repaired by NHEJ, which confirms the idea of transient DSBs which are not directly religated by Top2β. Hence, the Top2β-induced DSBs are long-lasting enough to unfold a physiological role in gene transcription, which is also confirmed by the increase of cFos expression, also in line with the role of Top2β-induced DSBs in neurons. However, it is worth mentioning that the risks of interfering with Top2β-mediated cleavage and rejoining as fundamental processes for regulated transcription are difficult to estimate, since the misrepair of such DSBs is considered to promote neurodegenerative diseases and carcinogenesis [9,10,11]. Nevertheless, our results provide a novel rationality for blocking Top2β activity in GBM therapy, extending the knowledge of NMDAR signaling pathways in GBM cells, which may also help to identify therapeutic targets in NMDAR signaling in other tumors expressing NMDARs.

## 4. Materials and Methods

### 4.1. Cell Lines and Cell Culture

The immortalized glioblastoma cell lines LN229 (IDH1^wt^) and U-87MG (IDH1^wt^; P53^wt^) were kindly provided by Prof. Franz Rödel (Frankfurt University Hospital, Frankfurt am Main, Germany). The cells were cultured in T75 flasks (Sarstedt, Nümbrecht, Germany) using DMEM (Sigma-Aldrich, St. Louis, MO, USA), supplemented with 10% FCS, 100 U/mL penicillin, 0.1 mg/mL streptomycin and 2 mM *L*-glutamine. The cells were not used for more than 15 passages.

The primary glioblastoma cell line G1702 was kindly provided by Prof. Donat Kögel (Frankfurt University Hospital, Frankfurt am Main, Germany). The G1702 cell line was established from a biopsy of a male patient and classified as a glioblastoma multiforme. The cells are IDH1^wt^, ATRX-positive and carry a hypermethylation of the MGMT promoter. The G1702 cells were cultured in T75 flasks (Sarstedt) as spheres in Neurobasal Medium (Gibco), supplemented with 2% B27, 100 U/mL penicillin, 0.1 mg/mL streptomycin, 2 mM L-glutamine, 20 nM EFG and 20 nM FGF2 at 37 °C, under a 5% CO_2_ atmosphere. We received the G1702 cell line at passage 30 and used it for no longer than 10 passages.

### 4.2. Transfection with siRNA

For siRNA transfection, 6 × 10^5^ LN229 cells were seeded in T25 flasks overnight and transfected with 16µg si-RNA (si1 UCGGGCUAGGAAAGAAGUAA(UU); si2 CAGCCGAAAGACCUAAAUACA(U U) eurofins; sequence described by Kamaci et. al., 2011 [43]) the next day, using the K2 transfection system (Biontex, Munich, Germany), and then incubated overnight. As a control, the cells were treated only with the K2 transfection reagent, but without RNA.

### 4.3. Immunofluorescence Staining

Next, 2 × 10^4^ LN229 or 4 × 10^4^ U-87MG cells were seeded in µ-slides VI^0,4^ (Ibidi) in a total volume of 150 µL/channel and directly treated as indicated. The next day, the cells were treated with 20 µM EdU for 30 min, fixed with 4% PFA, permeabilized with 0.1% Triton X-100 and stained with Hoechst 33342. EdU was detected with a Click-iTEdU imaging kit, following the manufacturer instructions (Thermo Fisher Scientific, Waltham, MA, USA), using Alexa Fluor 594 Azide (Thermo Fisher Scientific) and a 80 µL reaction buffer/channel. Then, the cells were blocked with 0.5% BSA/5% goat serum and incubated over night at 4 °C with primary antibodies: Rabbit anti-53BP1 (1:1000; H-300 Santa Cruz) and mouse anti-Top2β (1:100; A-12 Santa Cruz). The next day, the samples were washed three times for 10 min with PBG (PBS, 0.05% gelatin), then incubated with anti-rabbit/mouse Alexa Fluor 488/594 and labeled as a secondary antibody (1:400 Abcam) for 1 h at RT, then washed three times for 10 min with PBG and twice with PBS. Finally, the samples were imaged with the inverted epifluorescence microscope Axio Observer Z1 (Zeiss, Oberkochen, Germany).

The G1702 cells were stained with the same protocol with the following differences: The G1702 spheres were dissociated through repeated pipetting and accutase (Sigma-Aldrich) treatment for 1 to 2 min. Next, 5 × 10^4^ cells were seeded into µ-slides VI^0,4^ (Ibidi), coated with 2 µg/cm² laminin, and then stored overnight. The G1702 cells were not treated with EdU. The following primary antibodies were used: Rabbit anti-GluN1 (1:100, D65B7 Cell Signaling), mouse anti-GluN2B (1:200, S59-20 Stress Marq), rabbit anti-53BP1 (1:1000; H-300 Santa Cruz) and mouse anti-Top2β (1:100; A-12 Santa Cruz).

### 4.4. Analysis of 53BP1 Foci and cell cycle phases

For the foci counting and cell cycle analysis, the immunofluorescent stained samples were imaged with a 20× objective on the inverted epifluorescence microscope Axio Observer Z1 (Zeiss). Single nuclei were detected by the µManager software based on size and shape of the Hoechst 33342 signal. Then, the integrated density of the Hoechst 33342 signal of single nuclei was measured by µManager and blotted against their mean EdU signal. This blot allowed discrimination between the G1 phase cells with a low Hoechst 33342 and a low/no EdU signal, S-phase cells with an intermediate Hoechst 33342 and a high EdU signal, and G2-phase cells with a high Hoechst 33342 and low/no EdU signal. For the cell cycle analysis, a cell cycle phase of at least 1000 cells per experiment was determined (student’s *t*-test was used for statistics). For the 53BP1 foci counting, the non-S-phase cells or G1-phase cells were gated, depending on the experimental set up, then relocated and manually counted using a 63× objective. The foci of at least 40 single cells per condition and experiment were counted and the mean of all single cell values of all independent experiments were used for statistical analysis. For absolute foci values, the Mann–Whitney test (MWT) was used for statistics. For relative values, one sample *t*-test was used (GraphPad Prism 7.0, GraphPad Software, San Diego, CA, USA).

### 4.5. High-Content Microscopy

To count the 53BP1 foci in a high number of cells, we stained the cells as described previously in Section 4.3. 53BP1 was labeled with an Alexa 488 secondary antibody and EdU was labeled with Alexaazide 594. The samples were imaged via the Operetta High-Content Imaging System (PerkinElmer, Waltham, MA, USA) using a 40× high NA objective. The Harmony analysis software was used to select single nuclei based on the shape and intensity of the Hoechst 33342 signal. The EdU signal was used to exclude S-phase cells. Foci were automatically counted in the 53BP1 channel (using the “detect spot” feature). The same thresholds were set for all samples and at least 1500 cells per condition were counted. 

### 4.6. Western Blot

For the western blot analysis, 7 × 10^5^ LN229 cells were seeded in T25 culture flasks or 2 × 10^5^ G1702 cells were seeded in 6-well plates and treated overnight as indicated. On the next day, the cells were lysed in a 120 µL/40 µL ice cold lysis buffer (cell signaling #9803) containing protease inhibitor. Protein concentrations were determined using a BCA protein assay kit (Thermo Fisher Scientific) and ~30–60 µg protein was mixed with a 4× SDS-loading puffer (240 mM Tris/HCL pH 6.8, 40% glycerol, 8% SDS, 0.04% bromphenol blue) containing 100 mM dithiothreitol DTT, denaturized at 64 °C for 10 min and then and loaded on 6–12% gradient gel or 10% continuous gel per lane. The separated proteins were transferred to PVDF membranes in a semi-dry transfer system (BioRad, Hercules, CA, USA) for 36 min. The blots were blocked with 5% milk in TBS-T for 1h at RT, then treated with the rabbit anti-cFos antibody (1:2000; PA1318 Bosterbio), or the mouse anti-Top2b (1:500; A-12 Santa Cruz) or the rabbit anti-GAPDH (1:2000; FL-335 Santa Cruz) in 1% milk in TBS-T over night at 4 °C. Then, the blots were washed 3 times for 10 min with TBS-T and incubated with the anti-mouse/anti-rabbit HRP secondary antibody (Chemicon, Temecula, CA, USA) for 1h at RT, then washed again 3 times for 10 min. Immunoblots were detected using a luminol reagent (Thermo Fisher Scientific or Millipore) in the ChemiDoc MP imaging system (BioRad). Quantitative analysis was done with the Image Lab system (BioRad). All band intensities were normalized to the intensity of the GAPDH band in the same lane and the cFos/GAPDH ratios were normalized to the control treatment of the experiment. For statistics, one sample t-test was used (GraphPad Prism 7.0).

### 4.7. Clonogenic Survival

The LN229 cells transfected with or without Top2β siRNA were trypsinized for 24 h after transfection and seeded into 6 well plates as triplets and treated with 1 mMGlu. The untransfected cells were used as an additional control. The number of seeded cells was increased with the irradiation dose (siRNA transfected: 750 cells/well for 0 Gy and 2 Gy, 1500 cells/well for 4 Gy and 3000 cells/well at 6 Gy. Sham/untransfected: 500 cells/well for 0 Gy and 2 Gy, 1000 cells/well for 4 Gy and 2000 cells/well for 6 Gy). The cells were allowed to attach for 3 h and then irradiated in an X-ray tube with a tungsten anode (Philips, Amsterdam, Netherlands) at 33.7 mA and 90 kV with a dose rate of 1.162 Gy/min by Fricke dosimetry and at 45 cm distance using a 1 mm aluminum filter. Non-irradiated cells were placed in the radiation chamber for 100 s without irradiation. Colonies were allowed to form for 8 days, fixated with 70% ethanol and stained with 0.1% crystal violet in 25% ethanol. The colonies with more than 50 cells were manually counted using a binocular with 65-fold magnification and a 5 to 5 mm checkered counting grid to avoid double counting of single colonies. The plating efficiencies (PE) were determined depending on the number of seeded cells. The survival fraction was calculated by dividing the PE of each dose by the PE of the non-irradiated cells. The values of three independent experiments were fitted to a linear quadratic mathematical model by the GraphPad Prism software with the following equation: Surviving fraction (SF) = exp(−αD − βD^2^), where D is the X-ray dose.

## 5. Conclusions

In conclusion, our findings in the LN229 and primary GBM cells support a new approach for the therapy of brain tumors by targeting NMDAR-mediated Top2β-induced DSBs, which are required for regulating transcription. Thus, beyond the traditional function of the Ca^2+^-permeable NMDAR channels in excitatory neurotransmission and synaptic plasticity, these receptors are also proposed to play a role in human tumors by hijacking NMDAR signaling pathways, resulting in an increased expression of a subset of early-response genes which are implicated in tumor malignancy.
